# Making long‐acting treatment work: Tracing connections with extended‐release buprenorphine depot through time

**DOI:** 10.1111/dar.14021

**Published:** 2025-02-17

**Authors:** Sandra Gendera, Kari Lancaster, Tim Rhodes, Carla Treloar

**Affiliations:** ^1^ Social Policy Research Centre, UNSW Sydney Sydney Australia; ^2^ Centre for Social Research in Health, UNSW Sydney Sydney Australia; ^3^ University of Bath Bath UK; ^4^ London School of Hygiene and Tropical Medicine London UK

**Keywords:** extended‐release buprenorphine depot, long‐acting injectable buprenorphine, opioid agonist treatment, qualitative study, treatment retention

## Abstract

**Introduction:**

How people connect with opioid agonist treatment is an ongoing concern. Extended‐release buprenorphine depot (BUP‐XR) has been designed with ‘retention’ in mind. It is important to consider what makes a difference to clients in helping them to stay connected to treatment over time.

**Methods:**

We report findings from the third wave of in‐depth interviews with participants (*n* = 26) in the Community Long‐Acting Buprenorphine (CoLAB) study, tracing accounts of connection, disconnection and reconnection with BUP‐XR since initiation into treatment.

**Results:**

Changing situations in treatment delivery and in people's lives created conditions of possibility for connection and disconnection to treatment. Clients used BUP‐XR in different ways. Personalisation of dosing regimens and stretching out of time between doses was common, creating a sense of stability for some. For others, this flexibility potentiated fragility in treatment connection. Disconnection from BUP‐XR was common, but frequently this was not the ultimate outcome. Treatment connections were shaped by fluctuating life circumstances, with re‐connections imagined, attempted and sometimes realised.

**Discussion and conclusions:**

Clients' accounts reveal the complexities of how ‘long‐acting’ treatments are made to work over time. Connecting with treatment in the long‐term is a process, contingent on social relations, fluctuating life conditions and systems of care. Rather than treating connection and disconnection as opposites, we suggest seeing these as entangled and fluid elements of an ongoing process. What is needed is an adaptive and emergent conceptualisation of what ‘retention’ in treatment can mean, reflective of how people connect with their treatment and make it work, in practice.

## 1 INTRODUCTION

How people connect with opioid agonist treatment (OAT) is an enduring concern in treatment research and service delivery. Often defined in clinical terms as ‘treatment retention’, notions of connection constitute prime outcome measures of the effectiveness of treatments like methadone and buprenorphine, especially in the longer term. Research suggests that treatment access and affordability, flexibility of OAT provision, positive staff interactions, keeping drug cravings at bay and meeting patients' preferences, positively enhance treatment retention, whereas negative experiences, especially stigma, combined with restricted access and practices pose major barriers to retention [1, 2, 3, 4].

Newer treatment modalities such as extended‐release buprenorphine depot (BUP‐XR) are offering a broader range of treatment options and choices for people seeking treatment [5]. These long‐acting formulations have been designed with the issue of treatment retention as a core concern, obviating the need for adherence with daily dosing regimens or regular service attendance [6, 7]. To date, studies have examined the affordances of these new formulations, including how the shift away from daily dosing regimens is experienced by clients [8, 9, 10] as well as the changes to clinical practice accompanying their introduction into service [11]. Qualitative studies, in particular, have examined the multiple social and temporal effects of long‐acting OAT technologies and how time is made and stretched between doses [9, 10, 12]. As long‐acting formulations move from relative novelty to becoming embedded as standard practice, it is important to consider what makes a difference to clients in helping them to stay connected to treatment and care. How long‐acting formulations are made to work in the longer term is thus a key question. In this article, by tracing the interview accounts of participants engaged in an implementation trial of BUP‐XR in Australia, we examine how changing situations through time can create conditions of possibility for connection and disconnection to treatment. In doing so, we aim to generate new understanding of how long‐acting injectable buprenorphine formulations are made to work in practice in the long term.

## 2 BACKGROUND

In clinical trials, BUP‐XR treatments have been shown to be safe and effective [13, 14, 15, 16]. Studies have demonstrated high rates of retention and treatment satisfaction, reduction of non‐prescribed opioid use, and some improvements in social outcomes [7, 16, 17, 18, 19]. Participants administered with BUP‐XR for a year and longer maintained or improved their health, quality of life and employment participation [16, 17, 20, 21].

The novel treatment modality, designed to maintain consistent levels of buprenorphine in the bloodstream for up to a month at a time [5], entangles with socio‐environmental factors like time, convenience, cost and stigma attached to OAT [9, 10, 12, 22]. Combined, these characteristics of long‐acting injectable buprenorphine treatment are altering and challenging not only how OAT is conceptionalised and delivered within drug services [11] but also the perceptions, expectations and experiences of potential future and current recipients of these formulations [10, 12, 23].

A growing body of literature examining the two widely approved BUP‐XR formulations, Buvidal (for weekly and monthly administration) and monthly administration of Sublocade®, provides insights into the conditions shaping initiation and factors that influence retention in the early stages of treatment. Similar to initiating any new treatment, participants felt the need to understand the potential treatment effects [24, 25]. Treatment decisions were shaped by a mix of factors, including preferences for less than daily oral treatments, hopes of obtaining a different outcome through a new treatment, system limitations, including scarcity of alternative options, and the fear of not wanting to ‘miss out’ on this opportunity [23, 24]. Alignment of treatment expectations with embodied experiences and the extent to which the treatment was felt to ‘hold’ a person through the designated dosing period emerged as key considerations [10, 22, 23, 24, 25, 26, 27]. A gap between expected and embodied experiences could thus pose a risk to early treatment disconnection [10, 27, 28]. One longitudinal study by Parkin et al. [26], focused on participants who maintained BUP‐XR treatment continuously after initiation, characterised the treatment experience as a ‘journey’ in three stages, showing how participants gradually adjusted to the new treatment and built engagement over time. These findings offer insights into the early stages of BUP‐XR treatment journeys and suggest that longer‐term treatment connection was maintained because the long‐acting treatment met participants' expectations and treatment goals over time, keeping withdrawals and cravings at bay, while affording a wide range of social, health and personal benefits.

The literature also offers insights into when, how and why patients become disconnected from BUP‐XR. Not all participants starting, or maintaining BUP‐XR, see it as a longer‐term or indefinite ‘maintenance’ treatment. The design of the slow‐release formulation affords clients embodied and imagined opportunities to taper off and ‘become substance free’, embedding treatment disconnection in concepts of recovery [23, 28]. To date, the extant literature describes undesired and unexpected BUP‐XR treatment effects, creating or amplifying conditions for disconnection. Most notable undesirable or unexpected embodied effects included the dose not being sufficient for the person at the time (drug cravings, withdrawal symptoms), the treatment not working as anticipated (lumps, irritations, complications with other conditions) and a general sense of discomfort [23, 25, 28]. Beyond the directly experienced treatment effects, social and treatment‐system‐related conditions can create or accelerate discontinuation. BUP‐XR clients' treatment experiences of not being heard or validated, clients being ‘forced to switch’ to BUP‐XR [28], or being removed from the treatment due to ‘failure to adhere’ invite possibilities for disconnection [27]. Social and treatment‐system related conditions affecting treatment are not isolated to BUP‐XR and may affect treatment connection in spite of the treatment offered.

To date, the conditions of possibility that affect connections with BUP‐XR treatment have been researched in relatively narrow timeframes, also by separating out findings in groups of people ‘still on’ and those ‘who came off’ the treatment. The existing literature demonstrates how materiality (slow‐release depot and its technology), assumptions (promise of BUP‐XR), embodied effects (desirable/undesirable effects), affect (hopes, expectations) and service delivery arrangements and constraints come together in a situation to create varying outcomes. While these studies have been crucial for understanding patients' experiences and informing practice in the initial implementation phases, especially considering the novelty of BUP‐XR, they only provide a glimpse into the temporally contained experiences of BUP‐XR.

With the present study, we aim to extend and enrich understandings of how BUP‐XR is made to work in the longer term. We do this by tracing the experiences of a cohort of BUP‐XR participants (over three waves of longitudinal interviews, over 3 years) to understand how BUP‐XR is made to work through time. Through our analysis, we notice how the fluctuating situations that shape people's everyday lives create conditions of possibility for connecting with treatment in different ways. Instead of conceptualising treatment engagement as a binary or dichotomy – of being either ‘in’ or ‘out’, or ‘on’ or ‘off’, and with these states treated as respective indicators of ‘good’ or bad’ – we find that making treatment work in the long term is an emergent and fluid process. Connecting with treatment in the long‐term encompasses connections, disconnections and reconnections with treatment technology, with the form and strength of these connections oscillating over time in relation to everyday social situations. Our analysis helps to understand how long‐acting depot buprenorphine formulations are made to work in practice in the long term.

## 3 METHODS

### 3.1 Setting

The Community Long‐Acting Buprenorphine (CoLAB) study was a prospective single‐arm, multicentre, open‐label trial of monthly BUP‐XR. The study evaluated clinical outcomes among people with opioid dependence receiving BUP‐XR, as well as implementation practices across a range of healthcare settings in Australia. For full eligibility criteria and clinical procedures, see the CoLAB protocol [29], and for the clinical findings, see Refs. [16, 30].

### 3.2 Qualitative design

Embedded within CoLAB was a qualitative study. Here we report on findings emerging in the third wave of interviews, generated between October 2022 and April 2023, with our analysis situating these interview accounts within participants' longer‐term treatment experiences. Over 3 years, the study examined participants' accounts of receiving and living with BUP‐XR treatment, embodied experiences of the treatment, impacts on health, well‐being, relationships, participation in social life and employment, other drug use, service encounters and future treatment expectations.

The temporally situated conditions of the early stages of BUP‐XR treatment retention have been described elsewhere – including desirable, undesirable, or unexpected treatment effects, preference for different medications, the impact of relationships, trust and service delivery context [10, 22, 23, 24, 26, 27]. Our analysis of this third wave of interviews contributes to this literature by tracing participants' connections with treatment in the longer term, focused on how BUP‐XR was made to work within people's lives and different situations over time. The interviews also generated accounts of the service delivery context of BUP‐XR, including participants' experiences and perceptions of care, support and services through time, paying attention to the social, relational, environmental and policy constraints shaping individual treatment experiences.

### 3.3 Sampling and study sites

The interviews were undertaken by a single researcher (SG) across three time points to maintain continuity of relationships and contextual knowledge. Baseline interviews with 36 participants were conducted mostly in person (2019–2020) at sites of BUP‐XR administration or at people's homes and in public spaces. Subsequent interviews were conducted in person and by phone (due to restrictions related to COVID‐19), with 32 of the original participants followed up at wave two (2021) and 26 participating in phone interviews in wave three (October 2022 to April 2023).

Data were collected across four CoLAB sites, two in New South Wales and two in Victoria. The settings captured a diversity of Australian treatment service models across primary care and specialist clinics, as well as regional and metropolitan sites. All sites provided a suite of adjunctive interventions including psychosocial, case management and referral to rehabilitation services [10].

Of the original baseline sample (*n* = 36), 31 people had provided consent in year one to be recontacted for future research, beyond the completion of the clinical trial. At the time of the third wave of interviews, of the 31 consenting participants, five could not be followed up. Two declined their participation providing brief updates on their treatment journey; one person was in palliative care; two phone numbers were no longer in use and participants were not engaged with known services. Interviews lasted 40 min to 1 h and 50 min (on average 70 min long).

### 3.4 Recruitment and ethics

Participants were followed up using the last known contact details at wave two; services connected to the CoLAB study provided updated phone numbers where required. After sending personalised invitation text messages, participants were followed up over the phone to arrange a suitable interview time. The third wave of data collection stretched over 6 months; time and timing proved critical to the engagement and high follow‐up rate. The researchers ensured an interview environment that was sensitive to the place and events in a person's life, positioning them best to tell their story. Participants received a $AU54 cash/eftpos payment for their time.

The study had ethical approval from St Vincent's Hospital Sydney Human Research Ethics Committee (HREC/18/SVH/221) and was registered with ClinicalTrials.gov (NCT03809143). Verbal informed consent was recorded before the commencement of each interview.

### 3.5 Sample description

In wave three, we interviewed 26 participants aged 37–66 years; two‐thirds were male (*n* = 18) and eight were female (Table 1). Eleven participants had continuously received monthly BUP‐XR during the three‐year study period; while eight used different strategies and adaptations to make treatment work through time and in response to changing situations in their life, for example, by adjusting dosing intervals and engaging with the treatment in different ways.

**TABLE 1:** Community long‐acting buprenorphine participant characteristics from wave three interview.

Pseudonym	Age	Sex	Site	Continuously in receipt of BUP‐XR
Mark	52	Male	3	Yes
Damian	38	Male	4	Yes
Jack	49	Male	4	No
Melanie	55	Female	4	No
Jennifer	61	Female	4	No
Peter	66	Male	4	No
Kevin	37	Male	4	No
Terry	41	Male	4	No
Simon	45	Male	3	No
Adam	49	Male	3	Yes
Jacob	55	Male	2	No
Ken	47	Male	2	Yes
Miles	42	Male	2	Yes
Charles	42	Male	2	Yes
Edward	54	Male	2	No
Oliver	64	Male	2	No
James	45	Male	2	No
Henry	48	Male	2	No
Susan	51	Female	2	No
Annie	42	Female	4	Yes
Noah	40	Male	1	No
Betty	51	Female	1	Yes
Karen	47	Male	1	Yes
Donna	39	Female	1	Yes
Denis	43	Male	4	Yes
Carla	50	Female	4	No

Abbreviation: BUP‐XR, extended‐release buprenorphine depot.

### 3.6 Approach to analysis

Interviews were audio recorded and transcribed verbatim by a professional transcription service and deidentified using pseudonyms. For the first level analysis, we charted participants' stories using a framework method [31], in addition to generating snapshot accounts (1–2 pages long) for each participant to capture change over time [32]. The snapshot accounts mapped key quotes and treatment experiences together with contextual information surrounding these times of change and adaptations to treatment, drug use and expectations of BUP‐XR at the start, and long‐term treatment goals, tracing these through time. Accounts were iteratively analysed by two lead authors to identify, juxtapose and compare similarities and difference across the narratives. Third wave interviews focused on the emergent themes of participants' earlier accounts and traced how participants connected with treatment over time, including in the context of life experiences and social conditions.

Drawing on case examples from participants' accounts, we explore the *long* of long‐acting buprenorphine treatment as a process. Core themes in our analysis below include conditions affording ongoing treatment connections, conditions creating fluidity and fragility in treatment connections, treatment connections as shaped by fluctuating life circumstances, sudden life events, and treatment connections shaped by imagined, attempted and realised re‐connection.

## 4 RESULTS

Participants described a broad spectrum of treatment experiences across the study period.

### 4.1 Conditions affording ongoing treatment connections

Connection to treatment was made possible when treatment was felt to work with ease in participants' daily lives. Crucial to this was how the long‐acting formulation and its service delivery arrangements aligned with participants' own treatment and life goals.

Betty (51 years, Site 1) had tried several OAT treatments and months of residential rehabilitation. When the long‐acting treatment was offered, she hoped it would support her personal goals to stay opiate free. After 3 years being prescribed BUP‐XR, Betty was content and wanted to continue with BUP‐XR:

‘Like I've been to rehab. I've done all that, and I went back to my opiates, as soon as, you know, life got tough. This is why I refuse [to change my treatment]. This is why I didn't want to go off this injection and leave everything and think, ‘I'm okay now’, after three years. I didn't want to go through it, after all this hard work, and then fall again at my age.’ (Betty).

In the longer term, BUP‐XR offered Betty a feeling of safety and comfort. For Betty, the treatment met her expectations and worked ‘easily enough’ in her body. BUP‐XR was not felt to interfere with her daily life, work and family responsibilities, which allowed for the possibility of imagining longer‐term connection to treatment.

For Ken (55 years, Site 2), ongoing connection to BUP‐XR treatment was secured through the financial and social changes the treatment afforded him. The more flexible service delivery arrangements of long‐acting injectable buprenorphine created the ‘right’ conditions that enabled Ken, ‘for the first time in two decades’, to take up ongoing employment. When a friend made an offer of a well‐paid role with career prospects, Ken happily accepted. At the third interview, Ken had been working for 1.5 years, while learning a trade, he was also making concrete plans for a materially more secure future:

‘It just changed my life immensely! Like before, I was handcuffed to a chemist. Now, it's once a month, sometimes it's once every eight weeks […] just because I'm busy now, and I've got responsibilities at work and everything, I can't just go and use. Yeah. So, I've got to be clear minded because I go to trade school as well … Getting it is easy. [Dr name] sends me the script for the next month, it's on my phone … I go in [to get my depot injection] on a weekend. […] Now, I'm sort of trying to work towards, yeah, goals, like going on holidays and looking for property to buy, that sort of thing. My life is a lot different from where I was, you know, three‐four years ago.’ (Ken).

The long‐acting formulation fulfilled practical needs in the longer term, enabling Ken more choice and convenience around dosing and making it easier to combine a new busy work schedule with ongoing treatment, which in turn created the possibility to imagine different material futures in his life.

### 4.2 Conditions creating fluidity and fragility in treatment connections

The long‐acting characteristic of BUP‐XR, over time, enabled possibilities for remaining connected to treatment. Participants ‘personalised’ their use of BUP‐XR through different dosing approaches by stretching out of the time between doses. For some participants, these approaches were developed in collaboration with prescribers, while others trialled for themselves what worked at different times in their lives. Through personalisation possibilities for remaining connected to the treatment, it was made easier, including in situations that might otherwise have been disruptive.

At the same time, the flexibility of dosing intervals opened up potentials for connection to treatment to be made fragile, with ‘disconnection’ a looming possibility within these more generous constraints. Despite Ken's earlier account of the ‘ease’ of BUP‐XR, given his busy work schedule, Ken found that he was not regularly receiving doses every 4 weeks as scheduled. Ken described periods in which he would ‘stretch out’ the time between doses to 6 or even 8 weeks between injections:

‘[…] really, it just started out how busy I've been, you know, sometimes I'm also working six days a week, which just left Sunday. I couldn't be bothered getting out of bed to go down there early for my injection. So, I'm just, “Oh, bugger, I'll just go another week”. Because I wasn't feeling any withdrawal or any, you know, I wasn't feeling any worse for *not having* it. So, yeah, I felt at that time my rest and relaxation were probably more important than not catching up on any rest. […] For me personally it's probably a bit harder to get [the injection] … because I'm working full time. I see [the doctor] on Sundays, when they only do walk‐ins, there's no real appointments or scheduling.’ (Ken).

The flexibility of the service delivery arrangements (digital scripts, weekend and after‐hours opening times), combined with how the slow‐release agent produced embodied effects, enabled Ken, at times, to stretch out treatment doses as far as 8 weeks apart. Such oscillations afforded Ken greater choice but also weakened his connection to treatment. In his account, Ken repeatedly contemplated ‘stopping’ BUP‐XR, if only he could be certain he will ‘be alright for work’. With this speculation, Ken offered a glimpse of what the treatment may look like for him in the future:

‘Like it's really tempting to just to stop it altogether, you know. But with work, you know, I need to be at the top of my game. […] So, hopefully I can work something out with [my doctor] … and I can try and be free from the injections.’ (Ken).

Long‐term connection to treatment, for some, was an ambivalent or undesirable outcome. Not always did continuous connection to BUP‐XR align with participants treatment goals or indicate that the treatment ‘worked’ well for them. While some participants reported almost uniformly similar treatment effects across the 3 year study period (often described as feeling a kind of ‘nothingness’, see Ref. [10]), others experienced fluctuations of treatment effects with some swapping to different BUP‐XR treatments (including weekly formulations or different dosing) in an attempt to find the ‘right dose and treatment’ that would work for them. Donna (39 years, Site 1), a single parent, working a 50‐h week, said that about a year after initiating monthly‐BUP‐XR, during a highly turbulent time in her life, the treatment stopped working as well as it did at the start. Since then, over a year, Donna tried different doses, products and dosing intervals to find the treatment to ‘carry her over to the end of the month’:

‘In a good month, I don't even think about it. I don't think my injection is coming up. […] But [at the time], I was really, really stressed out, throughout the month, you know, something bad happened, yeah, it's just … it was almost like my body was eating it [BUP‐XR] up quicker in a way. […] For me, it's all about whether it works, for what it needs to work for… to carry me over to the end of the month, I don't want to feel bad when I'm at work or with my son.’ (Donna).

After experimenting in her second year with different BUP‐XR doses and products, Donna was content to have found ‘the right dose and treatment’. Simultaneously, Donna felt disappointed with the promise of BUP‐XR, having ‘hoped and expected’ the treatment would help her to taper off OAT easily, so she could ‘not be on anything […] not be reminded of my past’. As tapering off was still Donna's long term treatment objective, together with the clinical team, in the third year, she actively explored strategies to realise her goal of ‘living treatment free’.

### 4.3 Treatment connections shaped by fluctuating life circumstances

Participants' treatment expectations and goals influenced their interest and willingness to start and remain on extended‐release treatment. Equally influential, however, were the changing contexts of a person's life. How BUP‐XR was made to work in the long term was shaped by participants' (changing) situations, with possibilities for connection and disconnection continually in flux.

In the first few months, participants' imagined expectations of the treatment varied from their embodied experiences. For some, this disconnection between expectation and experience affected their decisions about continuing with BUP‐XR treatment. Some participants discontinued treatment altogether, while others chose different OAT options. Jennifer (61 years, Site 4) abruptly ended BUP‐XR after one injection, recalling feeling ‘disappointed’ and ‘surprised’ by the prominent and visible injection sites and pain receiving the injection. For 2 years after this, Jennifer remained connected to OAT by switching back to sublingual buprenorphine treatment. When Jennifer's personal situation unexpectedly changed, she was determined to find a treatment that could better support her during an especially unpredictable and socially unstable time. In her particular circumstances, and experiencing domestic violence, Jennifer wanted a treatment that would not keep her tied to a particular location or OAT service. Jennifer made the switch back to monthly BUP‐XR injections, giving an account of how her changing circumstances made the long‐acting treatment work for her in new ways, despite her previous ambivalence:

‘So, not only was I in this terrible domestic violence situation, also I had to be physically sick on top [because I couldn't get my sublingual dose for two days]. [Later] I was planning to go to rural Australia. […] That was the big headline, you know, “What am I going to do about my medication?” […] At the clinic, they said, “Well, there is another drug out, you can try this drug.” […] I found the [new BUP‐XR] injection suited me better. I only have to go to the clinic once a month instead of every couple of days, it is a life changing thing! […] I am able to hold down a job which is liberating. […] It's been a year that I am on monthly injections again; I go every 4 weeks. The clinic gives me my appointment in advance, […] my body is responding well to that so, I will just stick with what's working.’ (Jennifer).

A central theme in many participants' accounts was how unexpected life events drastically altered a participants' ‘journey’, often changing how a person had imagined or anticipated their ongoing connections with treatment. Events such as medical emergencies, moving interstate, falling in love, traumatic accidents, familial discord, domestic violence, homelessness and imprisonment entangled with, and shaped, what treatment could do. How BUP‐XR treatment was made to work for an individual changed with these shifting circumstances.

James (45 years, Site 2) received monthly BUP‐XR for 2 years, and after a year, he started to stretch out the dosing interval to 5–6 weeks, to accommodate busy family life. With guidance from the clinical team, James decided to taper off the treatment, gradually stretching time between BUP‐XR doses for even longer periods. During this time, James' family was involved in a car accident, adding pressure to daily living. It was then that James moved from BUP‐XR to sublingual, daily buprenorphine, to get the support he felt he needed:

‘So, exactly a year ago now, my wife and kids were in a car accident. So, that added to the pressure of life in terms of me doing more within my day. I mean my life's already busy. I've got three young kids. I run a business from home … [Around then] I was stretching out [monthly BUP‐XR] to around the 10‐ to 12‐week mark; it's when I started experiencing really bad opiate withdrawals. My doctor gave me [sublingual buprenorphine] if I needed to top up […] In the stress of everything that was happening in our life, I started to take Suboxone more […] before I knew it, I was taking the strips again daily. […] What I realised was, mentally I needed a daily medication at the time.’ (James).

The changing and fading of the appeal of BUP‐XR was another condition altering treatment connections, which could be accompanied by short‐ and longer‐term disconnections. Fading appeal related in some cases to participants' treatment goals shifting and changing over time, or the treatment being perceived as having ‘accomplished’ its intended objectives.

For some, disconnection from BUP‐XR was accompanied by feelings of ‘no longer needing the treatment’, manifesting through either a planned (guided by the medical team) or unplanned (‘ad hoc’ or circumstantial) tapering off to ‘be treatment free’. This tapering was often accompanied by curiosity about what ‘coming off’ a slow‐release treatment would feel like in the body, or alternatively a desire to try sublingual buprenorphine again to regain greater control over one's treatment. Jacob (57 years, Site 2) recalls the day he disconnected from BUP‐XR. For Jacob, it was ‘like I didn't need another one … I was going every 4 weeks, over two years […] then, one day I just didn't go back. [….] I hadn't planned it or anything, I just knew, it could have lasted me heaps longer.’ (Jacob).

### 4.4 Treatment connections shaped by imagined, attempted and realised re‐connection

By examining the situations of disconnection from BUP‐XR in participants' accounts, through time, we also notice narratives of imagined and attempted, and sometimes successful, reconnection to BUP‐XR treatment. Disconnection from BUP‐XR was often not the ultimate outcome for participants as they told their stories in the follow‐up interviews.

Susan (51 years, Site 2) wanted to be more in control of her treatment during stressful times in her life. After a year on monthly BUP‐XR, she moved to a weekly formulation, and later to daily sublingual buprenorphine. At the third interview, Susan explained her connection to the BUP‐XR treatment was ‘intermittent’, describing it as a ‘stop‐gap’ measure to affect/change heroin use when it ‘becomes too frequent and costly’:

‘I have the Sublocade, I've been having it intermittently. […] So, when I have the injection, I'm fine. Then I go back to using. Then I go back to the injection [BUP‐XR]. […] It's been a roller coaster. […] But it helps me during busy work periods.’ (Susan).

Treatment trajectories of people who disconnected and attempted to reconnect to BUP‐XR highlighted a mix of challenges, around BUP‐XR itself and the medical treatment systems not being set up to facilitate easy transition and reconnection. Susan hoped extended‐release treatment could afford her periods of non‐use, but achieving her envisioned BUP‐XR treatment objective proved challenging:

‘The last one [BUP‐XR injection] I had was in August this year, and I had it because I relapsed, but I had it too early … I actually went into severe instant withdrawals, I had it too quickly [after using heroin]. I was horrendously sick! It was terrible. […] Since then, I've been a bit scared, because I've been dabbling. Now I'm too scared to go and have the needle [BUP‐XR] again.’ (Susan).

Reconnection to monthly BUP‐XR treatment played out differently for Simon (45 years, Site 3), who was briefly (3 months) in receipt of BUP‐XR treatment after initial initiation. At the third interview, Simon says he recalled the treatment being ‘extremely effective’ the first time around. This embodied experience and past memory helped him envision his reconnection to BUP‐XR over other available treatment options. It was also due to dedicated wrap‐around support that Simon successfully reengaged in BUP‐XR treatment after 2 years of disconnection:

‘I was on the injection [3 years earlier], it was so effective, I couldn't believe it! It felt like I wasn't on anything at all […] I came off the injections, it was great … [in the second year] I went to jail for the first time in my life, I'd never been to jail before! […] Later, I met a girl that I really liked. She gave me an ultimatum […] or she was leaving. So, the only thing I knew, would work, was [BUP‐XR], that's why I went back, you know. I knew I could get clean straightaway; it would be effective … But my habit was huge, […] there's no way to be sober … I couldn't get back on it [BUP‐XR]. The girls [clinicians] up at the hospital, […] they knew me from previous [OAT] programs. […] When I rang, I explained, I was desperate, I wanted to get back on the injections … When they realised how many attempts I had made […], and it just wasn't happening […], they helped me. […] So, they just went the extra fucking mile, you know, and they came and got me [from the train station] drove me themselves to a detox facility and booked me in there and everything. They [OAT staff] were ringing every day to make sure I was alright; they were just brilliant!‘ (Simon).

## 5 DISCUSSION

How people can be supported to remain connected to OAT remains a matter of concern, including as newer long‐acting formulations become embedded into treatment systems [33, 34, 35]. The biomedical promise of long‐acting treatments is anchored in their potential to help overcome problems of treatment retention by removing the need for daily dosing [6]. Our findings highlight, however, that the potential of newer formulations is contingent not only on their long‐acting technology but also on how they are negotiated into social relations and fluctuating life conditions and systems of care. Innovative technologies such as BUP‐XR bring with them new, and at times surprising, effects for participants and for services [11, 36]. Monthly dosing is by no means an innovation to address the ‘problem’ of retention or cost efficiency [37, 38, 39]. Rather, the effects of long‐acting formulations are relational and contingent and are always, therefore, a *process*. Crucially, we find that participants' connections to treatment in the long term are not only contingent on their social arrangements but that the process of connecting with treatment over time is not a simple binary of being ‘in’ or ‘out’ of retention but a fluid relation in which there are different forms, strengths and styles of treatment connection and re‐connection.

This study examined how extended‐release monthly buprenorphine was made to work in the long term. We followed participants who connected, disconnected and reconnected with BUP‐XR treatment over 3 years since their initiation into treatment. The temporally situated conditions of the early stages of BUP‐XR treatment retention have been described elsewhere – including desirable, undesirable or unexpected treatment effects, preference for different treatment products, the impact of relationships, trust and service delivery context [23, 25, 26, 28, 40]. Our analysis traces participants' connections with treatment in the longer term, illuminating the multiple contingencies that constitute different forms of ongoing treatment engagement that extend beyond mainstream interpretations of ‘retention’. What supported participants to stay connected to treatment and care, or reconnect after periods of disruption, was complex, multifactorial and situationally created by fluctuating life circumstances and embodied concerns. We find that treatment connections – also involving disconnections and reconnections – are processes that are ‘made’ in their situations and over time, and that these arrangements extend beyond the effectiveness of particular treatments. While major life events could affect and alter how participants related to BUP‐XR treatment through time, these factors are not unique to BUP‐XR treatment and are known to affect treatment connection for other medical conditions.

In addition, we also found that the same long‐acting characteristics of the treatment itself could create conditions of possibility for clients to remain connected to treatment and care in the long term while bringing fragility and disruption to treatment trajectories for others. BUP‐XR formulations are known to afford flexibility and convenience for patients [9]. We observed how stretching out dosing intervals became a more regular practice and way of affording clients the opportunity to personalise their treatment, also enabling ways to attune treatment in the long term to minimise life interruption (see also, [10]). Participants, who were satisfied and expected to remain on treatment at year one, had left at follow‐up; others who disconnected early in the study, as the treatment did not meet their expectations, had re‐connected by the third wave interview. This is in contrast to conceptualisations of BUP‐XR trajectories as more linear, predictable ‘treatment journeys’ [26]. By following participants regardless of their treatment ‘status’ through the study, we noticed narratives of imagined and attempted, sometimes successful treatment reconnection after years of disruption, also ‘unorthodox’ applications of BUP‐XR as an intermittent or stop‐gap approach to disrupt non‐prescribed drug use. Disruption and disconnection from BUP‐XR was therefore often not the ultimate outcome for participants; these experiences need to be understood in their situation.

The long‐acting nature of BUP‐XR, allowing for adaptability and choice around dosing intervals at times experienced as the absence of treatment [10] could bring about desire and possibility for tapering off OAT altogether. Previous studies have hinted at the possibility of people exiting BUP‐XR, when they felt they ‘achieved’ their treatment goal [28]. Other studies have described the possibilities for engaging with BUP‐XR in an attempt to remake subjectivities rather than for long‐term maintenance [23, 24]. Our study confirmed these early findings. People connected to BUP‐XR in the long term often envisaged, imagined, considered, or in some cases, were successful in, tapering off BUP‐XR and OAT, either to realise a goal they had held to be ‘treatment free’, or as an objective emerging gradually in response to what they saw as the specific affordances of BUP‐XR. Tapering off or repurposing the treatment was sometimes done independently and experimentally, and in other cases proactively guided by clinical staff. The fading appeal of BUP‐XR and the material fragility of treatment ‘connection’ are possibly surprising findings, creating variable outcomes for clients and clinicians. The very feeling of ‘nothingness’ of treatment effect ([10], p. 112–13), which some describe as accompanying the stability and comfort of BUP‐XR, at times brings into question whether and how the treatment is not only working but whether it is needed.

The introduction of newer long‐acting formulations, like monthly BUP‐XR, invites a more nuanced and critical appreciation of how people connect with treatment in the long term, as well as what constitutes disconnection from treatment. Rather than treating treatment connection and disconnection as binaries or opposites, as mainstream ideas of retention invite us to do, our findings suggest seeing these as entangled and fluid elements of an ongoing process of how participants connect with treatment in the long term. The potential fragility of treatment connection emerging in the context of BUP‐XR, even as it is said to be working, also raises questions about how support and connection to care can be managed for people on long‐acting treatments [23], and for people ‘disconnected’, who may require non‐pharmaceutical interventions and support to achieve their treatment goals, or may want to reconnect to OAT at a later stage. For clinicians and services, these findings provide valuable insights into what helps clients remain connected to treatment and care in the long term, while understanding that the same conditions may lead to fragility or disconnection. These findings can inform the future design of services and support. Treatment connection, in our study, was supported by greater flexibility in service delivery and access to BUP‐XR (extended opening hours, weekend appointments, digital scripts to facilitate personalisation of dosing appointments and less restrictive monitoring practices). It was also supported by trust and quality relationships with staff, and staff acknowledging that practices like ‘stretching out’ dosing intervals were characteristic of this long‐acting formulation and might be expected in the long term. Responsive and adaptive approaches to treatment provision might be harnessed, rather than using punitive, restrictive practices to undermine attempts at treatment personalisation [27, 28]. While “small changes” to practice may seem marginal, as we have argued elsewhere, “tinkering as care – can make a profound difference to the relations of care” in OAT settings ([11], p. 9), helping people stay connected to treatment.

For people leaving OAT after receiving BUP‐XR, desiring, attempting, or effectively reconnecting to treatment requires its own set of practice considerations. Clear communication of ‘open‐door’ policies for people before they disconnect, where resources allow for, and ensure, clients have information and understand that ‘tapering off’ can become part of their treatment experience (planned or unplanned). Recognising how treatment is made to work in changing situations and lives also requires ameliorating conditions and practices that might work to produce stigma of ‘failure’ attached to clients' choice to reconnect to treatment and services, ensuring support is available and policies are flexible. Irrespective if people remain connected, or are disconnected from BUP‐XR, we see a need for different modes of social support, as well as information about and referral to alternative systems of support (peer groups, online and in‐person groups) to assist people who may want to remain connected to services, even while remaking themselves, for a time, or for longer, as ‘living treatment free’.

## AUTHOR CONTRIBUTIONS

Each author certifies that their contribution to this work meets the standards of the International Committee of Medical Journal Editors.

## CONFLICT OF INTEREST STATEMENT

The author(s) disclosed receipt of the following financial support for the research, authorship and/or publication of this article. This study was supported by an Externally Sponsored Collaborative Research grant from Indivior PLC. Indivior contributed to the study design and analysis plan of the clinical parent study [16] (Farrell et al., 2022) but not this qualitative study; Indivior had no role in collection, analysis and interpretation of data, in the writing of the manuscript or in the decision to submit the manuscript for publication. The Centre for Social Research in Health at the UNSW Sydney is supported by a grant from the Australian Government Department of Health. The views expressed in this publication do not necessarily represent the position of the Australian Government.

## ETHICS STATEMENT

The study received ethical approval from St Vincent's Hospital Sydney Human Research Ethics Committee (HREC/18/SVH/221) and was registered with ClinicalTrials.gov (NCT03809143). Verbal informed consent was recorded before the commencement of each interview.
